# The Pediatric Quality of Life Inventory™ (PedsQL™) family impact module: reliability and validity of the Brazilian version

**DOI:** 10.1186/1477-7525-6-35

**Published:** 2008-05-20

**Authors:** Ana C Scarpelli, Saul M Paiva, Isabela A Pordeus, James W Varni, Cláudia M Viegas, Paul J Allison

**Affiliations:** 1 Department of Pediatric Dentistry and Orthodontics, Faculty of Dentistry, Federal University of Minas Gerais – Av. Antônio Carlos 6627, Belo Horizonte, MG, 31270-901, Brazil; 2 Faculty of Dentistry, McGill University, 3640 University Street, Montreal, QC, H3A 2B2, Canada; 3 Department of Pediatrics, College of Medicine, Department of Landscape Architecture and Urban Planning, College of Architecture, Texas A&M University, 3137 TAMU – College Station, TX, 77843-3137, USA

## Abstract

**Background:**

Pediatric health-related quality of life (HRQOL) has emerged as an important health outcome in clinical trials and healthcare research, for which HRQOL assessment instruments have played an important role. However, these instruments are not available in all countries or all languages. The Pediatric Quality of Life Inventory™ (PedsQL™) Family Impact Module is a multidimensional instrument developed to assess the impact of chronic medical conditions on the HRQOL of parents and family functioning. The objective of the present study was to evaluate the psychometric properties of the PedsQL™ Family Impact Module cross-culturally adapted for use in Brazil.

**Methods:**

The PedsQL™ Family Impact Module was administered to 95 parents/guardians of children with cancer in active therapy from 2 to 18 years of age of both genders. Subjects were recruited by means of convenience samples from the Pediatric Hematology/Oncology Centers at two public hospitals. The 'in-patient' sample was defined as individuals who were hospitalized for the administration of chemotherapy. The 'out-patient' sample was defined as individuals who were receiving chemotherapy and were not hospitalized.

**Results:**

Test-retest reliability exhibited correlation values ranging from 0.81 to 0.96 for all subscales. Internal consistency reliability was demonstrated for the PedsQL™ Family Impact Module: Total Scale Score (α = 0.89), Parent Health-Related Quality of Life Summary Score (α = 0.83) and Family Summary Score (α = 0.73). The Total Impact Score for the in-patient and out-patient samples was 67.60 and 56.43, respectively (p < 0.01). The construct validity demonstrated that the PedsQL™ Family Impact Module proved capable of distinguishing between families whose children/adolescents were hospitalized and families of children/adolescents who are being taken care of at home.

**Conclusion:**

The Brazilian version of the PedsQL™ Family Impact Module was considered reliable and valid for assessing the impact of a chronic pediatric health condition on the HRQOL of parents and family functioning. The instrument should be field tested on other chronic pediatric illnesses.

## Background

Pediatric health-related quality of life (HRQOL) has emerged as an important health outcome in clinical trials and healthcare research. This is particularly true in the pediatric cancer population [1]. The significant progress in anti-neoplasm therapeutic protocols has enabled a reduction in mortality rates, especially in the last 40 years. Currently, many pediatric cancer patients can be cured if diagnosed and treated early. Therefore, there have been a growing number of studies aimed at assessing the HRQOL of pediatric cancer patients both during and following treatment. Decisions regarding the implementation of improvements in public healthcare may be adopted based on the impact of interventions on quality of life [1,2].

The impact of disease and treatment on family functioning plays an important role in a child's adaptation to chronic disease. The family's capacity to cope with the multiple sources of stress and uncertainty associated with their child's diagnosis and treatment is likely to affect the child's quality of life. The functioning and well-being of parents/guardians depend on the child's situation as well. Information on the quality of life of pediatric cancer patients and their families allows the identification of families with special needs for support or psychological intervention [3,4]. There is a vast range of coping strategies displayed by families in relation to both practical and emotional difficulties [5]. Childhood cancer affects individuals between 0 and 18 years of age and represents from 0.5 to 3.0% of malignant tumors in most populations. In Brazil, the estimated incidence of children with tumors in 2006 was 2.5% (11,800 individuals) of all cases of malignant growth or tumors caused by abnormal, uncontrolled cell division (malignant neoplasm) [6]. A better understanding of children and their families while coping with this specific stressor could be valuable to healthcare professionals.

In order to assess the impact of childhood cancer on the HRQOL of families, the decision was made to use the PedsQL™ Family Impact Module, which is a multidimensional instrument developed to assess the impact of chronic medical conditions on the HRQOL of parents and family functioning. The instrument was designed as a parent proxy-report instrument and can either stand alone or be integrated to the PedsQL™ Measurement Model [7,8].

The objective of the current study was to evaluate the psychometric properties of the PedsQL™ Family Impact Module cross-culturally adapted to Brazilian Portuguese.

## Methods

### Participants and Settings

This validation study was developed in the city of Belo Horizonte, Minas Gerais, Brazil. Subjects were recruited from the Pediatric Hematology/Oncology Centers at two public hospitals. This convenience sample included 95 families of Brazilian children and adolescents between the ages of 2 and 18 years of both genders, with malignant neoplasm and receiving chemotherapy. Thus, we selected individuals who were receiving medical care to induce remission [1]. The existence of another illness or concomitant syndrome to the malignant neoplasm was established as an exclusion criterion.

Proxy-reports were filled out by 95 parents/guardians who were interviewed at the hospital units. 'In-patient' status was defined as individuals who were hospitalized for the administration of chemotherapy and were always accompanied by a family member. 'Out-patient' status was defined as individuals who only came to the hospital for the administration of chemotherapy and were being taken care of at home. Most of the patients were in out-patient treatment. The in-patient sample (n = 29, 30.5%) was interviewed while hospitalized and the out-patient sample (n = 66, 69.5%) was interviewed while awaiting medical care. During the interviews, the parents/guardians also responded to a form regarding information on their age, kinship and degree of schooling. Interviews were performed individually by one of the researchers (ACS) in a room specifically reserved for this end. The interviewer restricted herself to reading the questions and answers of the questionnaire. Data collection took place between August 1, 2006 and December 20, 2006. Prior to the interviews, approvals were obtained from the Human Research Ethics Committees of the institutions involved. Written informed consent terms were also obtained from the participants.

### Instrument

The 36-item PedsQL™ Family Impact Module is a parent-report instrument designed to assess the impact of pediatric chronic health conditions on parents and the family. It includes 6 subscales measuring parents' self-reported functioning: Physical Functioning (6 items), Emotional Functioning (5 items), Social Functioning (4 items), Cognitive Functioning (5 items), Communication (3 items) and Worry (5 items); as well as 2 subscales measuring parent-reported family functioning: Daily Activities (3 items) and Family Relationships (5 items) [8]. The scale has five Likert response options, 'never', 'almost never', 'sometimes', 'often' and 'almost always' (corresponding to scores of 100, 75, 50, 25 and 0). Regarding the interpretation of the scale, higher scores indicate better functioning (less negative impact). The PedsQL™ Family Impact Module Total Scale Score is calculated as the sum of the 36 item scores divided by the number of items answered.

Two other scores can also be obtained. The Parent HRQOL Summary Score assesses the impact of cancer on the health-related quality of life of parents/guardians. The score is computed as the sum of the 20 item scores on the Physical, Emotional, Social and Cognitive Functioning Subscales divided by the number of items answered in these subscales.

The Family Functioning Summary Score assesses the impact of cancer specifically on family activities and relationships. The score is obtained from the sum of the 8 item scores on the Daily Activities and Family Relationships Subscales divided by the number of items answered in these subscales.

### Cross-cultural adaptation

Linguistic validation of the PedsQL™ Family Impact Module was performed following the PedsQL™ Measurement Model translation methodology [9,10]. The model of equivalence in the cultural adaptation of HRQOL instruments developed by Herdman et al. (1997) [9] was adopted for the planning, structuring and execution of the cross-cultural adaptation of the instrument [10].

The process was performed in five steps. In the first step, two translations from the original English-language instrument into Brazilian Portuguese were performed independently by two bilingual translators whose native language was Brazilian Portuguese.

In the second step, the two translated versions (T1 and T2) were analyzed by a group of specialists composed of 6 professionals from the field of Pediatric Oncology (one physician, three psychologists and two social workers). Special attention was given to the meaning of the words in the different languages (English and Portuguese) in order to obtain similar effects from respondents of different cultures. An effort was made to identify possible difficulties in understanding the questionnaire. A synthesis-version was developed (T3) as a result of this process.

The third step consisted of a backtranslation of the synthesis-version (T3) by a professional, bilingual translator whose native language was English. This translator had no access to the original instrument.

A subsequent comparison between the original version and the backtranslated version was performed by a third translator who was fluent in English and whose native language was Brazilian Portuguese. The fourth step was the analysis of semantic equivalence between the original and backtranslated questionnaires, assessed from the perspective of referential meaning of the constituent terms/words and general meaning of each item [9,11].

The concept of referential meaning was developed to evaluate the similarity between the literal meaning of the terms in the pairs of statements (original and backtranslated versions) [9,11]. Visual Analogue Scales were used for the analysis of referential meaning [12]. A single line with verbal and numeral descriptors at each end was constructed for each pair of statements (the original and adapted items). The Visual Analogue Scales were constructed with a horizontal line and vertical line anchors at either end labeled "complete meaning disagreement" and "complete meaning agreement", denoted as 0 and 100, respectively; the line was marked in units of 1 and labeled in units of 10 [13]. Thus, equivalence between the pairs of statements could be judged in a continuous form between 0 and 100%.

The concept of general meaning was used to evaluate the similarity regarding the idea transmitted by the statements between original and backtranlated versions. [9,11] A qualitative evaluation was carried out to assess item equivalence between the two versions. Each pair of statements was classified as: unaltered, slightly altered, very altered and completely altered.

The fifth step involved a preliminary qualitative evaluation of the proposed synthesis version. The PedsQL™ Family Impact Module was then applied to 20 individuals. In this phase, the interviewer carried out cognitive debriefing interviews in which the interviewees had the opportunity to suggest changes in words, phrases and expressions. They could also suggest examples for clarifying the question and express opinions on the acceptability, relevance and ease of comprehension of the questionnaire.

### Statistical analysis

Test-retest reliability was assessed using the Intraclass Correlation Coefficient (ICC) for total, summary and the 8 subscales scores. A 95% confidence interval was adopted. The ICC was measured according to the following values: ≤ 0.40 weak correlation; 0.41–0.60 moderate correlation; 0.61–0.80 good correlation; and 0.81–1.00 excellent correlation [14,15]. The Weighted Kappa Coefficient (kw) was also calculated for each question of the instrument to measure the degree of agreement for each pair of observations. The criteria considered in the interpretation of agreement: -1.0 to 0.0 poor; 0.0 to 0.20 discrete; 0.20 to 0.40 regular; 0.40 to 0.60 moderate; 0.60 to 0.80 substantial; 0.80 to 1.00 nearly perfect [16]. The PedsQL™ Family Impact Module was administered twice by the same researcher to 47 study participant families (49.5% of the overall sample), with a 7-day interval between occasions.

Internal consistency was determined using Cronbach's Alpha Coefficient. Values ≥ 0.70 were considered acceptable for comparisons between groups [17,18].

Construct validity was established using the "known-groups method". We hypothesized that families whose children/adolescents were hospitalized would report higher scores (less negative impact) than families whose children/adolescents were being taken care of at home. In Brazil, continuous health care in a hospital has a distinctive importance for families, with continuous access to physicians, nurses, medication and a balanced diet [19]. In order to determine the magnitude of the differences between families, effect sizes were calculated. This analysis was calculated by taking the difference between the in-patient mean and the out-patient mean divided by the pooled standard deviation. Effect sizes for differences in means are designated as small (0.20), medium (0.50) and large (0.80) [20].

Data analyses were carried out with SPSS statistical software (version 12.0).

## Results

### Sample characteristics

The instrument was applied to 95 parents/guardians of children/adolescents with malignant neoplasm; 66 (69.5%) of the parents/guardians were related to individuals in the out-patient sample. Table 1 displays a descriptive analysis of the demographic characteristics of the overall sample. Most of the children/adolescents had been diagnosed with leukemia (55.8%). The average age of the parents/guardians was 35.4 years (standard deviation = 9.7); 76.8% were mothers and 62.1% had up to 8 years of schooling.

**Table 1:** Descriptive analysis: demographic characteristics of the sample

**Demographic characteristics**	**Total sample (N = 95)**	
	N	%
	
**Children/adolescent characteristics**		
**Ages (years)**		
2–4	33	34.8
5–7	16	16.8
8–12	28	29.5
13–18	18	19.0
**Gender**		
Boys	60	63.2
Girls	35	36.8
**Cancer diagnosis**		
Leukemia	53	55.8
Lymphoma and reticuloendothelial neoplasm	16	16.8
Others	26	27.4
**Groups**		
Out-patient	66	69.5
In-patient	29	30.5
		
**Characteristics of parents/guardian**		
**Ages (years)**		
20–40	73	76.8
41–79	22	23.2
**Relationship to patient**		
Mother	73	76.8
Others	22	23.2
**Level of schooling**		
≤ 8 years	59	62.1
> 8 years	36	37.9

In order to assess the test-retest reliability, the instrument were administered for a second time to 47 (49.5%) of the 95 parents/guardians one week following the first interview. The health condition of the children was clinically similar between the first and the second interviews.

### Cross-cultural adaptation

During the cross-cultural adaptation, the group of specialists stated that the concept of the impact of childhood cancer on the quality of life of the families used for development of the original instrument was pertinent to Brazilian culture. The assessment of semantic equivalence was performed between the items from the backtranslated synthesis-version and the items from the original version. Considering the referential meaning, 86.1% of the 36 items exhibited "complete meaning agreement", as rated on a Visual Analogue Scale. The general meaning remained unaltered in 86.1% of the pairs of statements. The interviewees reported that they enjoyed answering the questions and considered the research very important. The parents did not report any problems in understanding the instructions and response choices of the instrument. However, they made a number of suggestions for replacing words and expressions.

### Construct validity

The construct validity of the PedsQL™ Family Impact Module was determined by comparing scores obtained by the parents/guardians from the in-patient and out-patient samples. Table 2 displays means, standard deviations, analysis of effect sizes and t-test results of the responses on each subscale of the PedsQL™ Family Impact Module in the in-patient and out-patient samples. The effect size ranged from medium to large for the all of the subscales except "Cognitive Functioning" and "Daily Activities".

**Table 2:** Scale descriptors for the PedsQL™ Family Impact Module: comparisons between in-patient and out-patient samples

		**In-patient sample**			**Out-patient sample**				
**Subscale**	**Number of items**	**N**	**Mean**	**SD**	**N**	**Mean**	**SD**	**Difference**	**Effect Size**

**Total Impact Score**	36	29	67.60	13.53	66	56.43	16.27	11.17***	0.75
**Parent HRQOL Summary Score**	20	29	72.20	13.86	66	62.18	17.07	10.02***	0.65
									
Physical Functioning	6	29	70.55	20.41	66	58.23	23.55	12.31**	0.56
Emotional Functioning	5	29	68.62	15.69	66	55.08	20.35	13.54***	0.75
Social Functioning	4	29	76.94	22.60	66	69.29	26.28	7.65	0.31
Cognitive Functioning	5	29	74.48	25.72	66	68.79	22.21	5.69	0.24
Communication	3	29	72.99	29.18	66	61.49	26.18	11.50	0.42
Worry	5	29	48.28	26.74	66	33.18	19.68	15.09***	0.65
									
**Family Summary Score**	8	29	67.46	21.77	66	56.25	22.35	11.21*	0.51
									
Daily Activities	3	29	48.85	35.55	66	49.50	32.15	-0.64	-0.02
Family Relationships	5	29	79.31	23.89	66	59.24	26.86	20.07***	0.79

N = number of individuals; SD = standard deviation.

*p < .05, **p < .02, ***p < .01; equal variance was not assumed for Social Functioning and Worry subscales.

### Reliability

The test-retest reliability analysis of the PedsQL™ Family Impact Module scales is displayed in Table 3. The subscales exhibited excellent ICC values (>0.80). Agreement of the items revealed Weighted Kappa Coefficient values of 0.31–0.85, thereby ranging from regular to nearly perfect.

**Table 3:** PedsQL™ Family Impact Module: Test-retest reliability (N = 47)

**Subscale**	**Sample (N = 47)**
**Total Impact Score**	0.96 (0.92–0.97)*
**Parent HRQOL Summary Score**	0.95 (0.92–0.97) *
Physical Functioning	0.90 (0.82–0.94) *
Item 1: ...tired during the day	0.53^#^
Item 2: ...tired in the morning	0.55^#^
Item 3: ...feel too tired to do things	0.55^#^
Item 4: ...headaches	0.65^#^
Item 5: ...body weakness	0.60^#^
Item 6: ...nausea	0.77^#^
Emotional Functioning	0.81 (0.66–0.90) *
Item 1: ...anxiety	0.31^#^
Item 2: ...sadness	0.56^#^
Item 3: ...angry	0.54^#^
Item 4: ...disappointment	0.49^#^
Item 5: ...helplessness and hopelessness	0.65^#^
Social Functioning	0.93 (0.87–0.96) *
Item 1: ...isolation	0.68^#^
Item 2: ...difficult to get help	0.85^#^
Item 3: ...difficult to find time to have fun	0.60^#^
Item 4: ...lack of energy to have fun	0.61^#^
Cognitive Functioning	0.92 (0.85–0.95) *
Item 1: ...difficult to pay attention to things	0.63^#^
Item 2: ...difficult to remember what people tell me	0.46^#^
Item 3: ...difficult to remember what I have just heard	0.61^#^
Item 4: ...difficult to think quickly	0.60^#^
Item 5: ...difficult to remember what I was just thinking	0.64^#^
Communication	0.81 (0.65–0.89) *
Item 1: ...people do not understand my family's situation	0.45^#^
Item 2: ...difficult to speak about my child's illness	0.38^#^
Item 3: ...difficult to tell the doctors and nurses how I feel	0.61^#^
Worry	0.91 (0.83–0.95) *
Item 1: ...worry whether my child's treatment is working	0.66^#^
Item 2: ...worry about the side effects of medications	0.57^#^
Item 3: ...worry about how people will react to the illness	0.47^#^
Item 4: ...worry about how the illness affects my family	0.63^#^
Item 5: ...worry about my child's future	0.71^#^
**Family Summary Score**	0.95 (0.90–0.97) *
Daily Activities	0.89 (0.80–0.94) *
Item 1: ...family activities takes more time and effort	0.54^#^
Item 2: ...difficult to find time to finish the household chores	0.60^#^
Item 3: ...fatigue made it difficult to finish the household chores	0.64^#^
Family Relationships	0.85 (0.73–0.92) *
Item 1: ...lack of communication between people in my family	0.60^#^
Item 2: ...conflicts between people in my family	0.56^#^
Item 3: ...difficult to make group decisions in my family	0.69^#^
Item 4: ...difficult to solve family problems	0.73^#^
Item 5: ...stress and tension between people in my family	0.55^#^

*p ≤ 0.001(2-tailed) Intraclass Correlation Coefficient (ICC) – Confidence Interval 95%

^#^Weighted kappa Coefficient (kw) was calculated for each item

Table 4 displays the internal consistency reliability alpha coefficients for PedsQL™ Family Impact Module subscales. The Total Impact Scores, the Parent HRQOL Summary Score and the Family Summary Score achieved values greater than 0.70 in the total, in-patient and out-patient samples. However, some subscales presented values near or below 0.70 when assessed separately, the lowest (0.52) achieved on 'emotional functioning' subscale in the in-patient sample. The 'emotional functioning' and 'social functioning' subscales achieved Cronbach's alpha coefficients between 0.52 and 0.67 in the total, in-patient and out-patient samples.

**Table 4:** Internal consistency reliability: Cronbach's Alpha Coefficient on the Brazilian version of the PedsQL™ Family Impact Module for total, in-patient and out-patient samples

**Subscale**	**Total Sample (N = 95)**	**In-patient sample (N = 29)**	**Out-patient sample (N = 66)**
**Total Impact Score**	0.89	0.86	0.89
			
**Parent HRQOL Summary Score**	0.83	0.78	0.83
Physical Functioning	0.70	0.64	0.69
Emotional Functioning	0.62	0.52	0.59
Social Functioning	0.65	0.61	0.67
Cognitive Functioning	0.75	0.84	0.69
Communication	0.60	0.72	0.52
Worry	0.70	0.78	0.58
			
**Family Summary Score**	0.73	0.76	0.70
Daily Activities	0.69	0.77	0.66
Family Relationships	0.81	0.81	0.78

The internal consistency reliability alpha coefficients for the Brazilian version and original English version of the PedsQL™ Family Impact Module are presented in Table 5. Both the original and Brazilian versions achieved Cronbach's alpha coefficients greater than 0.70 in the total, in-patient and out-patient samples.

**Table 5:** Internal consistency reliability: Cronbach's alpha coefficient on the Brazilian and original versions of the PedsQL™ Family Impact Module for total, in-patient and out-patient samples

		**Cronbach's α coefficient**		
**Scale**	**Items^a^**	**Total sample**	**In-patient sample**	**Out-patient sample**

**Total Impact Score**				
Brazilian version	36	0.89	0.86	0.89
Original Version	36	0.97	0.97	0.95
				
**Parent HRQOL Summary Score**				
Brazilian version	20	0.83	0.78	0.83
Original Version	20	0.96	0.96	0.95
				
**Family Summary Score**				
Brazilian version	8	0.73	0.76	0.70
Original Version	8	0.90	0.93	0.89

## Discussion

Care for children with cancer should encompass supporting and helping families to cope with all aspects of treatment, including the diagnosis and the uncertainty of the outcome. Psychological distress following a diagnosis of childhood cancer involves risks of long-term psychosocial problems for parents and families. High rates of depression or posttraumatic stress symptoms are reported. Frequently, the entire family experiences disruption in their daily routine. In order to help families adjust positively to the illness, the assessment of the heath-related quality of life of parents and families could contribute toward their healthcare needs [3,21,22].

This study presents the reliability and validity of the Brazilian Portuguese version of the PedsQL™ Family Impact Module, a multidimensional instrument developed to assess the health-related quality of life (HRQOL) of parents and family in the context of childhood cancer. HRQOL measurements have been the target of research investigations in the healthcare field and a number of HRQOL assessment instruments have been developed. However, these instruments are not yet available in all countries or languages. Most questionnaires have been developed in English-speaking countries and adapted for use in other countries [23-25]. Considering the differences between social, cultural and economic aspects, the availability of cross-culturally valid, multi-lingual versions of instruments is important for obtaining reliable, comparable data [26].

The cross-cultural adaptation of the PedsQL™ Family Impact Module was performed following a specific protocol (PedsQL™ Measurement Model translation methodology) [27], which ensures the adoption of a single methodology for the adaptation of the scale in different countries. Regarding the assessment of the semantic aspects, it was concluded that the pairs of translation/backtranslation statements achieved adequate equivalence vis-à-vis the original questionnaire. The involvement of the group of specialists should be emphasized, as they contributed with reflections and discussions, thereby promoting suitable adjustments in the developed synthesis-version.

Instruments should produce similar results in two or more administrations to the same individual, provided that the general clinical state has not been altered [24]. The analysis of test-retest reliability suggests the adequate stability of the PedsQL™ Family Impact Module. The 7-day interval between interviews was important in diminishing the probability of systemic alterations in the clinical condition of the individuals. It is recommended that the interval between measurements be long enough to reduce the effects of memory and short enough to diminish the likelihood of systemic alterations. Although the definition of this interval is arbitrary, a period of 2 to 14 days is considered adequate [12,24,28,29].

Considering the internal consistency of the PedsQL™ Family Impact Module, the Cronbach's α coefficients exceeded the recommended minimum of 0.70 for the total impact score and the summary scores, demonstrating the adequate homogeneity of the scale. As in the original version, the Brazilian version performed reliably. However, values were heterogeneous when assessing each subscale separately. The 'emotional functioning', 'social functioning' and 'communication' subscales should be used disjointedly only for descriptive or exploratory analyses, as they did not achieve a alpha coefficient of 0.70 in the total sample. A study carried out in San Diego and Los Angeles (USA) with 339 families of individuals with cancer between the ages of 2 and 18 years found alpha coefficients of less than 0.70 in various subscales of the versions designed for children/adolescents [30]. This low internal consistency may be related to the small number of items that compose the subscales as well as the small sample size [30,31]. Furthermore, alpha coefficient values may be influenced by the low level of schooling in the sample [25,32].

Construct validity was evaluated using the differentiation of groups that are known to be distinct [1,25,33,34]. The data demonstrated statistically significant differences between families whose children/adolescents are hospitalized and families of children/adolescents who were being taken care of at home. The hypothesis established was supported: families whose individuals are hospitalized have higher functioning than those whose children are living at home. Therefore, the occurrence of illness implied limitations and difficulties in the functioning of the entire family. This fact was also reported in a study with 23 medically fragile pediatric patients in San Diego, United States [8].

Except for the "Daily Activities" subscale, the means obtained in all other subscales were greater in the in-patient sample, confirming that childhood cancer in hospitalized individuals had a lesser negative impact on family functioning than in those living at home [8,21].

This study has certain limitations that should be recognized. The generalizability of the findings is limited by two factors: the small sample size and the selection of a specific chronic pediatric condition. Sample size is an ever present difficulty in studies on individuals afflicted with cancer, stemming from the low prevalence of the illness [25,29,32,35]. In order to broaden this convenience sample, the study encompassed the two largest childhood cancer treatment hospitals in Belo Horizonte, Brazil. Although the sample size decreased the probability of detecting significant differences, 7 of 11 comparisons between in-patient and out-patient samples were statistically significant regarding the different scales and subscales. Further studies should be conducted to test the performance of the instrument on groups of children with other chronic health conditions. It should also be stressed that the scale was developed to be self-administered. However, due to the low level of schooling among the individuals of the study, the option was made to administer the questionnaire in interview form in all cases. A number of studies have demonstrated that the mode of administration does not affect the performance of the instruments [25,33-37]. Nevertheless, a comparison between the interview mode of administration and self-filled out mode of administration needs further investigation. In the present study, there was no report by the parents/guardians of any lack of comprehension regarding the questions.

## Conclusion

The Brazilian version of the PedsQL™ Family Impact Module exhibited adequate properties regarding the reliability and validity of the construct. This suggests its usefulness as a parameter in studies assessing the impact of pediatric cancer on the HRQOL of parents and family functioning. The PedsQL™ Family Impact Module should be field tested on other chronic pediatric illnesses in order to permit the generalization of the findings.

## Abbreviations

PedsQL™: Pediatric Quality of Life Inventory™; HRQOL: Health-Related Quality of life; ICC: Intraclass Correlation Coefficient.

## Competing interests

The authors declare that they have no competing interests.

## Authors' contributions

ACS, SMP, IAP, JWV and PJA conceptualized the rationale and design of the study, ACS and CMV performed the statistical analysis and interpretation of the data, ACS, SMP and PJA drafted the manuscript. All authors read and approved the final manuscript.
